# Tropane alkaloids GC/MS analysis and low dose elicitors’ effects on hyoscyamine biosynthetic pathway in hairy roots of Algerian *Datura* species

**DOI:** 10.1038/s41598-018-36625-4

**Published:** 2018-12-18

**Authors:** Boualem Harfi, Lakhdar Khelifi, Majda Khelifi-Slaoui, Corinne Assaf-Ducrocq, Eric Gontier

**Affiliations:** 1 Ecole Nationale Supérieure Agronomique (ENSA), L-RGB, El-Harrach, Alger Algeria; 2 Centre de Recherche en Biotechnologie (C.R.Bt), Ali Mendjeli, Constantine Algeria; 3 Université Picardie Jules Verne (UPJV), UFR des Sciences, Amiens, France

## Abstract

Plant secondary metabolites play a major role in plant adaptation to stress. Species belonging to *Solanaceae* family and *Datura* genus produce tropane alkaloids: *D. stramonium*, *D. tatula* and *D. innoxia*. These species are cultivated for their hyoscyamine (HS) content, whence the interest of this study to induce transformed roots of these species with strain A4 of *Agrobacterium rhizogenes*. Hairy roots (HRs) of *Datura* were established at high frequency by infecting vitroplants. All HRs (343 independent lines) were next employed to study the production of HS and growth. A screening of HRs alkaloid content by GC/MS is performed; it reveals, for the first time, the production of 13 alkaloids by the selected root lines. The selection of high productive line offers an interesting option to enhance the productivity. As HS is the dominant compound, the lines of *Datura* species were selected for their characteristics for biomass and HS production. The elicitors salicylic acid (SA) and acetyl salicylic acid (ASA) were also used to increase HS production. The results showed that the optimal concentration of the two elicitors (AS and ASA) was 0.1 mM. The highest HS content (17.94 ± 0.14 mg g^−1^ D.W.) obtained in HRs of *D. tatula* treated with 0.1 mM of acetyl salicylic acid.

## Introduction

The family of *Solanaceae* represents a very important source for secondary metabolites such as alkaloids. Used in medicine, alkaloids are considered to be anticholinergic drugs model. Pharmaceutical industry manufactures active pharmaceutical substances containing tropane moiety in their structure, which are applied as mydriatics, antiemetics, antispasmodics, anesthetics and bronchodilators^1^. From the *Solanaceae*, the *Datura* species are particularly rich for their tropane alkaloid content among which hyoscyamine (HS) and scopolamine were the main compounds because of their operation on the human nervous system^2^. Several species of genus *Datura* such as *Datura tatula* L., *Datura stramonium* L. and *Datura innoxia* Mill. show genetic diversity in relation to the alkaloid content^3^. Most of these products derived from wild plants. However, in most cases, the productivity is too low for an economically feasible production and the interest for HS is always in increase^4^.

Classical and most used strategies to improve secondary metabolites productivity of plant cell and tissue cultures are three. The first one is the screening and selection of high producing line; the second is the optimization of growth and production media and the last one is based on the induction of secondary metabolite production by abiotic or biotic elicitors and metabolic engineering.

Hairy roots (HRs) obtained by genetic transformation with *Agrobacterium rhizogenes* have been extensively used for *in vitro* production of HS and scopolamine^5^. They seem to be the most promising approach for feasible alkaloid production. They are characterized by a reasonable genetic and biosynthetic stability of selected transgenic line. It is thus of interest to be able to improve secondary metabolite production by means of genetic engineering^6^. So, after induction of HRs, the first step consists on screening of high producing root lines^7^.

Elicitation is a strategy for enhancing the metabolites in the adventitious root cultures of many plants. Since the biosynthesis of secondary metabolites is controlled during development and the metabolites are accumulated in response to abiotic stresses or attack of pathogens, stress-signaling molecules are frequently used in elicitation experiments. Elicitors are generally considered to modulate many morphological and physiological events in plants^8,9^. These remains a need to increase alkaloid production rates to favor their commercial exploitation^10^. Among the various elicitors, salicylic acid (SA) and acetyl salicylic acid (ASA) are used for improve the alkaloid production in transgenic roots^11,12^. SA is stress-signal molecules related to the activation of plant defense genes and to the biosynthesis of secondary metabolites^8^.

In this communication, we report the establishment of stable hairy root cultures of three species belonging to the genus *Datura*, obtained by infection with *Agrobacterium rhizogenes* strain A4. These transformed root cultures were screened for their high alkaloids production and their biomass.

GC/MS approach has been proved being an effective analysis method for alkaloid identification and quantification in *Datura* genus^13,14^. In this study, we report the tropane alkaloid spectrum of HRs of three Algerian *Datura* species.

In addition, works on elicitation of alkaloid production showed that the increase in the production was accompanied by a loss of biomass^11,15,16^. Present study was focused on the improvement of HS production by HRs using two elicitors (AS and ASA). Elicitors used at low concentrations and with relatively important elicitation time in order to increase HS production and to avoid biomass losses.

Finally, selected lines were analyzed after treatment with SA and ASA for their tropane alkaloid metabolism. Factors influencing root growth, biomass and HS production are evaluated and discussed.

## Results

### Screening of hairy root lines

**Figure 1:**
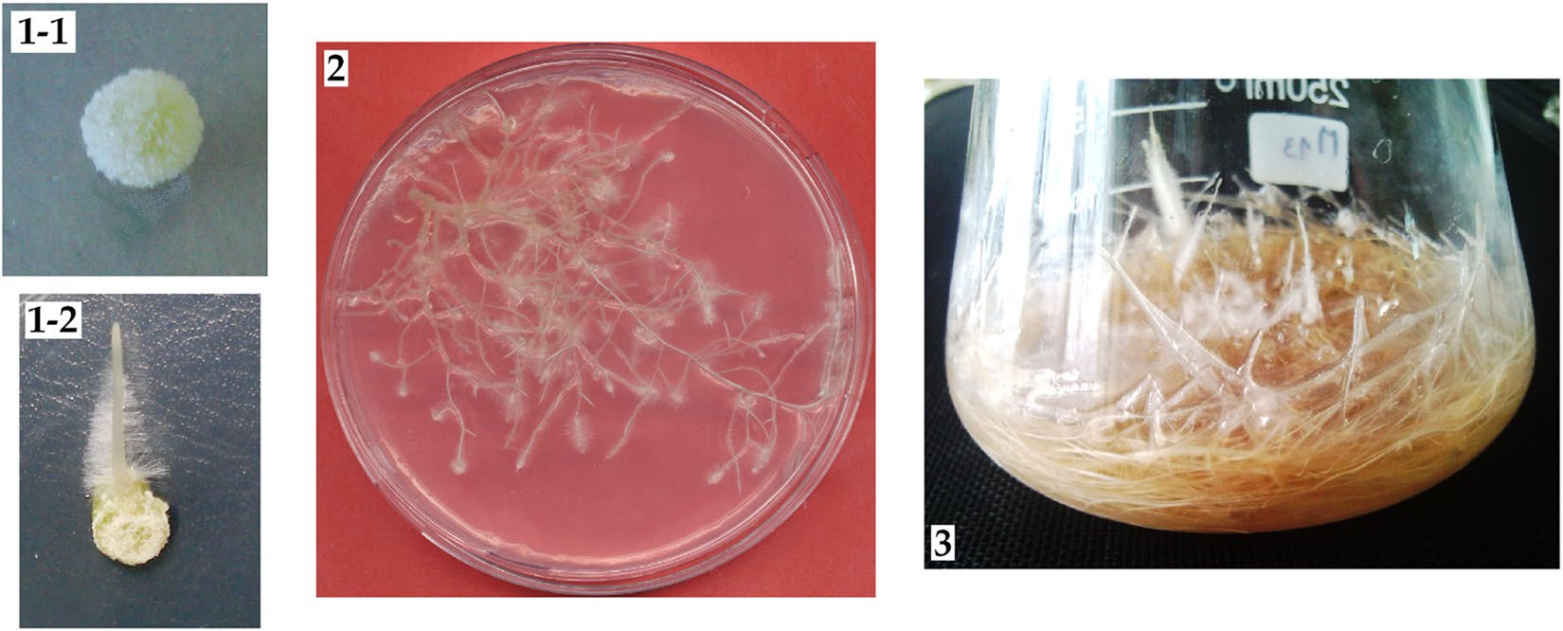
Hairy roots induction, multiplication and growth. 1-1: callus formation on the inoculation site 1-2: hairy root at the infection sites of *Datura* explant (fragment of hypocotyl); 2: selected hairy root line (multiplication on B5 solid medium); 3: selected hairy roots growth on B5 liquid medium.

In total 343 HR lines were generated and three lines were selected, one for each species. The first selection criterion was lateral and longitudinal growth. Root’s length, ramification’s degree (number of secondary and tertiary roots relative to the main root), biomass (Fig. 1) and HS production were observed and measured. The characteristics of the three selected root lines after 20 days of culture on B5^17^ liquid medium were described by Harfi *et al*.^16^.

### GC/MS analysis for no elicited root lines

**Figure 2:**
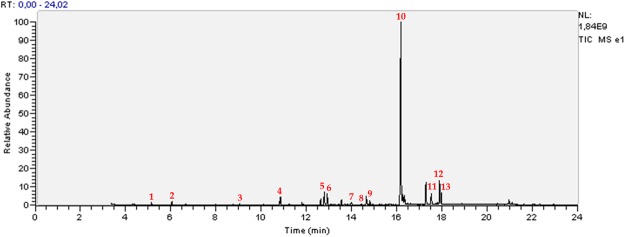
Gas chromatography of the identified tropane alkaloids in hairy roots of *Datura tatula*.

**Figure 3:**
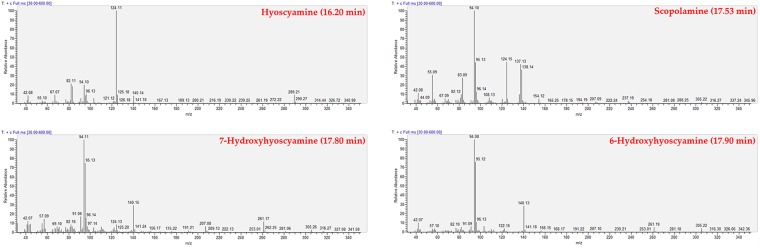
Mass spectra of major compound (hyoscyamine) and three other tropane alkaloids.

In our study, GC/MS analysis of the three non-treated *Datura* selected roots lines detect the presence of 13 tropane alkaloids (Table 1; Figs 2 and 3), HS being the most important.

**Table 1:** Tropane alkaloids identified, by GC/MS, in hairy roots of three *Datura* species from Algeria.

Peak	Retention time (min; ±0.05)	Experimental RI	Bibliographic RI	M+/base ion (*m/z*)	Alkaloids name	Molecular Formula	References
01	5.14	1100	1058	141/84	Hygrine	C_8_H_15_NO	^13^
02	6.05	1208	1167	141/82	Tropine	C_8_H_15_NO	^13^
03	9.08	1414	1465	199/82	Methylecgonine	C_10_H_17_NO_3_	^27^
04	10.83	1531	1655	223/124	3α-Tigloyloxytropane	C_13_H_21_NO_2_	^13^
05	12.80	1927	1825	199/94	3-Acetoxy-6-hydroxytropane	C_10_H_17_NO_3_	^13^
06	12.90	1934	1830	239/113	3α-Hydroxy-6β-tigloyloxytropane	C_13_H_21_NO_3_	^13^
07	14.00	2058	1937	259/124	3-Phenylacetoxytropane	C_16_H_21_NO_2_	^28^
08	14.44	2091	2000	309/94	3-Tigloyloxy-6-isobutyryloxytropane	C_17_H_27_NO_4_	^13^
09	14.83	2145	2020	271/124	Apoatropine	C_17_H_21_NO_2_	^28^
10*	**16.20**	2284	2170	**289/124**	**Hyoscyamine**	C_17_H_23_NO_3_	^13^
11	17.53	2403	2292	303/94	Scopolamine	C_17_H_21_NO_4_	^13^
12	17.80	2459	2335	305/94	7-Hydroxyhyoscyamine	C_17_H_23_NO_4_	^28^
13	17.90	2464	2355	305/94	6-Hydroxyhyoscyamine	C_17_H_23_NO_4_	^28^

RI: Kovats retention index.

*Bold: Major compound

### Effects of SA and ASA elicitation on hyoscyamine production

As HS the dominant compound, the study of elicitation focused on the possible ways to stimulate its production in the three selected root lines using different concentrations of the two elicitors.

Table 2 shows the results obtained after elicitation of HR lines previously selected for the three species of *Datura*. The variance analysis shows a significant effect of salicylic acid (SA) and acetyl-salicylic acid (ASA) on HR’s HS content. The Tukey’s test revealed 5 homogenous plots for L_DS_, 6 for L_DT_ and 7 for L_DI_.

**Table 2:** Effects of various concentrations of salicylic acid and acetyl salicylic acid on hairy roots growth and hyoscyamine content for three species of *Datura*.

Root lines	Elicitor	Concentration (mM)	D.W. (g)	HC (mg/g D.W.)	Improvement factor	
					$\frac{{\rm{D}}{\rm{.W}}{\rm{.}}\,{\rm{after}}\,{\rm{elicitation}}}{{\rm{D}}{\rm{.W}}{\rm{.}}\,{\rm{without}}\,{\rm{elicitation}}}$	$\frac{{\rm{HC}}\,{\rm{after}}\,{\rm{elicitation}}}{{\rm{HC}}\,{\rm{without}}\,{\rm{elicitation}}}$
L_DS_	C	0	0.205 ± 0.002^b–e^	8.16 ± 0.61^CD^	—	—
	SA	10	0.173 ± 0.010^d–f^	6.84 ± 0.50^D^	0.84	0.84
		1	0.195 ± 0.006^d–f^	7.84 ± 0.25^D^	0.95	0.96
		0.1	0.206 ± 0.004^b–e^	**10.58 ± 0.43** ^**AB**^	1.00	**1.30**
		0.001	0.203 ± 0.003^e–f^	9.30 ± 0.62^BC^	0.99	1.14
	ASA	10	0.128 ± 0.003 ^f^	2.87 ± 0.53^E^	0.62	0.35
		1	0.182 ± 0.006^d–f^	7.56 ± 0.44^D^	0.89	0.93
		0.1	0.199 ± 0.002^d–f^	**11.58 ± 0.17** ^**A**^	0.97	**1.42**
		0.001	0.204 ± 0.003^b–e^	11.48 ± 0.48^A^	0.99	1.41
L_DT_	C	0	0.295 ± 0.006^a^	8.57 ± 0.44^E^	—	—
	SA	10	0.246 ± 0.010^a–d^	9.65 ± 0.64^E^	0.83	1.13
		1	0.277 ± 0.004^a–b^	11.41 ± 0.73^D^	0.94	1.33
		0.1	0.299 ± 0.006^a^	**16.78 ± 0.21** ^**B**^	1.01	**1.96**
		0.001	0.296 ± 0.006^a^	14.01 ± 0.24^C^	1.00	1.63
	ASA	10	0.235 ± 0.006^a–d^	4.01 ± 0.23^F^	0.80	0.47
		1	0.274 ± 0.005^a–c^	13.43 ± 0.16^C^	0.93	1.57
		0.1	0.297 ± 0.003^a^	**17.94 ± 0.14** ^**A**^	1.01	**2.09**
		0.001	0.300 ± 0.005^a^	14.34 ± 0.15^C^	1.02	1.67
L_DI_	C	0	0.229 ± 0.004^a–d^	3.15 ± 0.13^EF^	—	—
	SA	10	0.191 ± 0.006^d–f^	2.66 ± 0.40^F^	0.83	0.84
		1	0.203 ± 0.003^c–e^	3.82 ± 0.13^E^	0.89	1.21
		0.1	0.239 ± 0.002^a–d^	**6.61 ± 0.09** ^**C**^	1.04	**2.10**
		0.001	0.231 ± 0.004^a–d^	5.75 ± 0.30^D^	1.01	1.82
	ASA	10	0.185 ± 0.005^d–f^	1.76 ± 0.22^G^	0.81	0.56
		1	0.204 ± 0.004^d–f^	3.57 ± 0.18^E^	0.89	1.13
		0.1	0.233 ± 0.004^a–d^	**8.89 ± 0.29** ^**A**^	1.01	**2.82**
		0.001	0.236 ± 0.004^a–d^	7.68 ± 0.22^B^	1.03	2.44

Bold characters represent the best results; the values are given as the mean ± SD; L_DS_: selected root line from *Datura stramonium*; L_DT_: selected root line from *D. tatula*; L_DI_: selected root line from *D. innoxia*; C: control; SA: salicylic acid; ASA: acetyl salicylic acid; D.W.: dry weight; HC: hyoscyamine content; Indice and capital letters: statistical classification of HS contents; Indice and minuscule letters: statistical classification of dry weight.

Addition of 0.1 mM ASA resulted in gave the maximum yield of HS for the selected line of *D. stramonium* (L_DS_) after 24 h of exposure (11.58 ± 0.17 mg g^−1^ D.W.) and increased the HS content up to 1.42-fold compared with the control over the same period (8.16 ± 0.61 mg g^−1^ D.W.). The best results with SA were obtained with always concentration of 0.1 mM. However, the transgenic root growth of *D. stramonium* was not affected significantly by SA and ASA treatments at the concentrations of 0.1 and 0.01 mM (Table 2). Nevertheless, when the cultures were treated with 10 mM ASA, the root biomass decreased to 38% compared to the control culture.

The HS content of HRs of the selected line with *D. tatula* (L_DT_) reached maximum (improvement factor > 1) with 0.1 mM of SA or ASA with respectively 16.78 ± 0.21 mg g^−1^ D.W. and 17.94 ± 0.14 mg g^−1^ D.W. Also ASA at 10 mM leads to a decrease of HS content and reduced HS to 4.01 ± 0.23 mg g^−1^ D.W. given an improvement factor of 0.47-fold. Neither SA nor ASA, at the concentrations of 0.1 and 0.01 mM, influenced biomass accumulation in the transformed root culture. Finally, after treatment with 0.1 mM SA or ASA, the best HS content for HRs of *Datura innoxia* (L_DI_) is 6.61 ± 0.09 and 8.89 ± 0.29 mg g^−1^ D.W. respectively (improvement factors: 2.10-fold and 2.82-fold). The root growth was not affected by these treatments.

In the present study, the effect of SA or ASA on the production of HS was optimal at 0.1 mM without HRs biomass loss. The addition of the elicitors SA and ASA at 10 mM affected significantly the root growth of the three selected lines.

## Discussion

To obtain high alkaloid-producing root clones, systematic selection, based on growth rate and alkaloid content, is necessary because the induced HRs lines exhibited different growth and individual biochemical properties in this study. The majority of HRs showed a fast growth. Nevertheless, some transgenic roots were characterized by a weak growth and/or callus formation. These root lines were systematically eliminated.

Published studies on tropane alkaloids content of *Datura* HRs report the production (detection) of only two alkaloids, namely, hyoscyamine and scopolamine^2,3,11,15^. In this study, GC/MS analysis of no treated root lines shows the presence of 13 tropane alkaloids, HS being the major compound. El-Bazaoui *et al*.^18^ reported the identification of nine tropane alkaloids in the whole plant of *D. stramonium* by GC/MS. This study is the first to reveal the biosynthesis of 13 alkaloids in HRs of the three species of *Datura* genus.

SA and ASA are involved in signal transduction and induce the transcription of biosynthetic enzymes involved in the formation of defense compounds in plants^19^. The general cellular process and regulatory principle for activation of plant secondary metabolite biosynthesis is that, an extracellular or intracellular signal is perceived by a receptor on the surface of the plasma membrane or endomembrane; the elicitor signal perception initiates a signal transduction network that leads to activation biosynthesis of transcription factors, which regulate the expression of biosynthetic genes involved in plant secondary metabolism^8^. The HRs of *Datura sp*. were treated with SA or ASA for 24 h with low different concentrations, after incubation, the biomass and HS content were determined and compared to the untreated control.

The effectiveness of ASA and SA elicitors used as a tool to enhance the production of alkaloids has been described for different species^20,21^. Elicitation has been developed as a promising alternative for *in vitro* producing metabolites by plant cell cultures^8^. SA is also a signal molecule in so-called induced systemic resistance (ISR) but SA is not referred as an universal inductor in the formation of defense compounds in plants. Nevertheless, recently the role of SA in the regulation of secondary metabolites like as indole alkaloids or tropane alkaloids has been reviewed^10,20^.

Ajungla *et al*.^15^ grew the root cultures developed from *Datura metel* leaves in B5 medium supplemented with 1.2 µM IAA for study the effects of biotic and abiotic elicitors on root growth and HS and scopolamine production. They reported that the highest HS levels were 1.39 (mg g^−1^ D.W.) in the no-elicited control and 4.35 (mg g^−1^ D.W.) in the roots elicited with SA (0.5 mM) with an improvement rate of 3.13-fold.

When the HR cultures were treated with methyl jasmonate, yeast extract or oligogalactoronids, the improvement factor of tropane alkaloids in HRs is function of elicitor, with the following order: methyl jasmonate, yeast extract and oligogalactorunids. The same study showed that methyl jasmonate affected the HS rate in HRs; compared to control without elicitation the authors recorded an improvement factor of HS content of 1.82-fold^11^.

Generally, elicitation influences the root growth and the biomass in the transformed root culture. When the cultures of adventitious roots of *Scopolia parviflora* were treated with 2.0 mM methyl jasmonate, the root growth index (ratio between the dry weight of the harvested root and dry weight of initial inoculum) decreased to 63% of the control culture after 72 h of exposure^10^. Zabetakis *et al*.^11^ reported that the treatment of *Datura stramonium* HRs, with 0.1 µM methyl jasmonate, reduced fresh weight to 11.3% of the control. Similarly results were reported with jasmonic acid in *Datura stramonium* HRs^22^. The elicitation with NaCl (from 17.24 to 172.4 mM) and Na_2_SO_4_ (from 7 to 70.42 mM) increasing concentrations improves HS and scopolamine accumulation into adventitious roots of *D. metel* but decreases significantly the growth index of root cultures^15^. However, Kang *et al*.^10^ reported that SA at low levels did not have a negative effect on growth of adventitious roots of *Scopolia parviflora* and did not stimulate root browning unlike methyl jasmonate.

Ours results confirmed these conclusions. Treatments with 0.1 and 0.01 mM SA and ASA enhanced HS production without effect on HRs biomass compared to the control. Nevertheless, high concentrations of SA and ASA (10 and 1 mM) lead to a biomass decrease to 38% for *Datura stramonium* line. This result agrees with those reached for some other plants, e.g. adventitious root of *Scopolia parviflora* elicited with 2 mM of methyl jasmonate, a loss of a third of root biomass (dry weight) was recorded, however, treatment with 0.1 mM SA or 0.1 mM of methyl jasmonate does not affect root growth^10^.

Amdoun *et al*.^22^ reported HRs of *Datura stramonium*, after 28 days of culture with HS production of 2.1 and 3.8 mg g^−1^ D.W. into B5 medium and optimized B5 medium respectively. In the case of jasmonic acid elicitation, the HS content was 4.2 and 8.5 mg/g D.W. in the HRs grown respectively in B5 medium and optimized B5 medium but the HR biomass was decreased. Our results show that the HS content of *Datura stramonium* HRs (control) is 8.16 mg g^−1^ D.W. after 20 days of culture into B5 medium. The HS production of this line, after ASA elicitation, is 11.58 mg g^−1^ D.W. Nevertheless, the highest content is 17.94 mg g^−1^ D.W. for HR line of *Datura tatula* after elicitation with ASA (0.1 mM) without biomass inhibition.

Demand for natural products has been continuously increasing because of their significant value, mainly as pharmaceuticals. Aseptic systems for biotechnological and large-scale production of plant-derived secondary metabolites are significantly improved. These molecule of interest bio-production systems are attractive tools because of their stability. Plant secondary metabolism evolved in the context of highly organized and differentiated cells and tissues, featuring chemical complexity operating under tight environmental^4^.

The results obtained in Table 3 show the significant effects of ASA and SA elicitation on HS production. For the three species of *Datura*, elicited HR abundances (mg g^−1^ D.W.) were compared to those not elicited and to the bibliographic data by Shimomura *et al*.^23^, Boitel-Conti *et al*.^24^ and Harfi *et al*.^3^. We noted that the ASA elicitation (0.1 mM) which has been used with the *Datura tatula* HRs enhanced HS accumulation 6.08-fold compared to bibliography. The highest improvement factor (2.82-fold) was observed with the HRs elicited with ASA (0.1 mM) for the *Datura innoxia* lines compared to the control. Our experiments with ASA (0.1 mM) are another example for the significant role of elicitation to induce the production of HS. The present study shows that the highest HS improvement rate after elicitation (1.32 to 2.82 -fold) corresponds to literature. Nevertheless, the HS contents of our selected HR lines are already high, so the improvement rates are between 1.89 to 6.08 fold.

**Table 3:** Effects of salicylic acid and acetyl salicylic acid on hyoscyamine content (HC) of hairy root lines of three *Datura* species compared to control (C) and bibliography (B).

Species	Elicitor (0.1 mM)	HC (mg/g D.W)	Values of controls (mg/g D.W.)		Improvement factor HC after elicitation/HC of control	
			Experimental HRs No elicited (C)	Bibliography HRs (B)	Experimental (C)	Bibliography HRs (B)
DS	SA	10.58 ± 0.43^B^	8.16	5.6^23^	1.30	1.89
	ASA	11.58 ± 0.17^B^			1.42	2.07
DT	SA	16.78 ± 0.21 ^A^	8.57	2.95^3^	1.96	5.69
	ASA	17.94 ± 0.14 ^A^			2.09	**6.08**
DI	SA	6.61 ± 0.09 ^C^	3.15	1.9^24^	2.10	3.47
	ASA	8.89 ± 0.29 ^C^			**2.82**	4.67

Bold characters represent the best improvement factors compared to the experimental controls and the bibliography; C: Hyoscyamine content (HC) of no-elicited Hairy Roots (HRs); B: HC of HRs from bibliography; DS: *Datura stramonium*; DT: *D. tatula* and DI: *D. innoxia*.

The HRs are generally a heterogeneous population which the individual lines show various genetic and physiologic characteristics. Therefore, the screening of HRs is a prerequisite to obtain high producing lines. Effectively, Yukimune *et al*.^25^ and Harfi *et al*.^16^ demonstrated that the systematic selection could be a tool for the production of high producing lines of secondary metabolites. This can be explained by the fact that obtaining a large number of root lines increases the probability of retaining, after selection, an interesting HR with good secondary metabolite and biomass production.

In summary, our study confirmed the role of SA and ASA into HS induction in HRs of three *Datura* species without biomass decrease. The role of SA on tropane alkaloids elicitation maybe explained by the fact that SA is a stress-signal molecule, naturally synthesized in the plant cell under stress and tropane alkaloids are the response to the micro-environmental changes. The elicitation of HRs from selected lines with ASA (0.1 mM) improves significantly the HS content. The HS rates of 11.58 ± 0.17, 17.94 ± 0.14 and 8.89 ± 0.29 mg g^−1^ D.W. have been obtained with *D. stramonium*, *D. tatula* and *D. innoxia* without biomass inhibition. Also, in this study, we detect, by GC/MS analysis, 13 tropane alkaloids produced by HR controls of the three *Datura* species.

## Methods

### Plant material

Three species of *Datura* were used: *D. stramonium* L., *D. tatula* L. and *D. innoxia* Mill. *Datura* sp. seeds were collected in Mitidja (North of Algeria), scarified and surface disinfected^16^. They are sown in test tubes containing 20 ml of solid hormone-free Murashige and Skoog (MS) medium (Sigma, USA) containing 2% sucrose and 0.7% agar. They are cultivated at 26 °C ± 1 °C with a daily 16 h photoperiod. For each species, two plots were grown, 48 vitro-plants were used for each plots. The hypocotyls fragment from 0.5 to 1 cm were taken on the *Datura sp*. vitro-seedling 45 days old.

### Establishment of hairy root cultures

Hairy roots (HRs) cultures were obtained via genetic transformation as described by Harfi *et al*.^3^ with *Agrobacterium rhizogenes*. The used A4 strain of *A. rhizogenes* is an agropine-type plasmid. After the inoculation of bacteria on solid medium (activation of *A. rhizogenes* conserved à 4 °C), they were put in suspension in the YEM (Yeast Extract Medium) liquid culture medium for 72 hours (approximately 10^6^ germs/ml). The co-culture (bacteria – explant) was done by simple deposit using a syringe on the level of the basal section of the hypocotyls fragments, from 0.5 to 1 cm, taken on the *Datura sp* vitro-seedling two months old. The culture of hypocotyls is operated on MS solid medium containing 250 mg L^−1^ cefotaxime. Only emergent roots from the infection site were retained.

Four or five weeks after inoculation, the first HRs appeared at the infection sites of explants. Root segments were excised when they reach approximately 2 cm length and transferred into MS solid medium containing 250 mg L^−1^ cefotaxime. After several passages onto B5 medium for elimination of the bacteria, the HRs were placed on fresh B5 solid medium without antibiotic and were cut off in 2 cm long segments.

Induction HRs was estimated by the induction rate of HRs corresponding to a percentage of vitro-plants with transgenic roots at the infection site, the time of the first hairy root apparition and the mean of transgenic roots for each vitro-plant.

### Screening of effective hairy root lines

The selection of efficient hairy root lines realized according to the protocol described by Harfi *et al*.^16^. Hairy roots that exhibited good growth (length of the main root greater than 15 cm after 20 days of culture on solid B5 medium) were retained and selected according to three main criteria: average speed of growth (cm per day), hairy root dry weight (D.W. [g]), and average levels of hyoscyamine (mg g^−1^ D.W.) after 20 days of culture. Selection of efficient root lines was carried out in Petri dishes containing hormone-free B5 medium supplemented with 7 g L^−1^ agar and 20 g L^−1^ sucrose placed in culture chamber at a temperature of 26 ± 1 °C and in total darkness.

### Alkaloids extraction

The extraction for all samples carried out on 50 mg of dry matter^22^. HRs were dried for 48 h at 40 °C, powdered and then, 50 mg were extracted with 6 mL of hexane for 5 min. The hexane phase containing fat compounds (but not alkaloids) was then discarded. 12 mL of HCl 0.1 N were then added for 10 min and after centrifugation, NH_4_OH (28%) was used to reach pH 10. Aqueous phase was filtered and extracted three times with an equal volume of chloroform. Organic phase was then dried with anhydrous Na_2_SO_4_. After another filtration step, the organic phase was evaporated and the residue was suspended again in 5 ml of dichloromethane, filtrated at 0.2 µm^22^.

### GC/MS (screening of tropane alkaloids content) and GC (determination of hyoscyamine for treated root lines) analysis

For tropane alkaloids screening of selected root lines, the filtrates were analyzed by GC/MS according to the protocols of Nguyen *et al*.^26^.

GC/MS analysis was conducted on a Trace GC Ultra instrument, equipped with a Triplus autosampler and coupled to a DSQ II mass spectrometer (Thermo Fischer Scientific, Waltham, USA) as described in Nguyen *et al*.^26^. Alkaloids were analyzed using a low-bleed VF-5 MS column (30 m × 0.25 mm × 0.25 µm) (Varian Inc., Grenoble, France). One microliter was injected in splitless mode at 250 °C. The temperature of the oven was kept at 40 °C for 1 min, increased by 30 °C min^−1^ to 130 °C and then by 10 °C min^−1^ to 280 °C and kept at 280 °C for 5 min. Helium was used as carrier gas at a flow of one mL min^−1^. Temperatures of transfer line and ion source were 300 °C and 200 °C. Mass spectra were recorded with a scanning range of 30–600 *m/z*, at 4.7 scans s^−1^ ^26^.

Peak finding, peak integration and retention time correction of the GC/MS chromatograms were performed with the XCMS Rpackage. Integrated peaks of the mass (*m/z*) fragments were normalized across all samples by expressing the peak areas relative to both an internal standard in each sample (ribitol or homatropine), and the exact dry weight of tissue used in each extraction. The XCMS output of integrated peaks was tested for robust integration^26^.

Each tropane alkaloid was identified by comparison of its mass spectrum to the NIST05 database and to the internal database of the laboratory issuing from Nguyen *et al*.^25^. The retention index of each compound was checked before final annotation. The level of each compound was estimated as % area based on the surface of peaks measured in total ion current^26^.

For treated root lines, the content of HS was analyzed by Gas Chromatography. The GC was performed on a CHROMPACK CP 9002 (Chrompack International B.V., Middelburg, The Netherlands), equipped with a FID detector. The dry residue was dissolved with CH_2_Cl_2_ and filtered. A fractionated sample (2 µL) was injected into a capillary DB1 column (Agilent, 30 cm × 0.32 mm internal diameter). The GC conditions were as follows; Azote (N) as carrier gas; detector temperature, 260 °C, injection temperature, 260° and oven temperature, 250 °C. Combustion gas was hydrogen.

Hyoscyamine identification and quantification by GC were performed using a standard prepared under the same conditions as the samples. To make the calibration curve, solution of the standard dissolved in CH_2_Cl_2_ was prepared with four concentrations (0.2, 0.4, 0.8 and 1.6 mg ml^−1^). This was repeated three times.

### Elicitation

Salicylic acid (SA) and acetyl salicylic acid (ASA) were obtained commercially from Sigma (St. Louis, MO, USA). Each solution was prepared by dissolving elicitor in an adequate volume of distilled water and sterilized.

The root segments 30 mm long from the selected lines were cut off and transferred into the 50 mL of the liquid medium. The medium consisted of B5^17^ medium supplemented with 2% sucrose and adjusted to pH 5.8 before autoclaving. The hairy root cultures were established by inoculation of 0.1 g (fresh weight) of roots into 250 mL Erlenmeyer flasks. The flasks were maintained under darkness at 26 ± 1 °C on an orbital shaker at 70 rpm. The elicitors were added at concentrations of 10, 1, 0.1 and 0.01 mM into the flasks cultivated for 19 days. Non-treatment is referred to control (HRs lines in B5 liquid medium without elicitor). The elicitation effect was measured at 24 h after adding SA and ASA. The dry weight (D.W.) of HRs and the HS content were estimated and compared with control and literature. The biomass (fresh and dry weights) of HRs and their content in HS were defined after 20 days of culture. Drying is carried out with 60 °C during 48 h.

### Statistical analysis

Statistical analysis was carried out according to variance analysis (ANOVA) and Tukey’s test for the obtained results. The values followed by the same letter are not significantly different from each other (p: 0.05).
